# Development of a Sustainable Biocatalytic Process for Geranyl Benzoate Production Using Immobilized Lipase

**DOI:** 10.1002/open.202500476

**Published:** 2026-02-12

**Authors:** Domenico Meola, Chaimae Chaibi, Simona Aprile, Karina Cesca, Debora de Oliveira, Ariela Veloso de Paula, Francesco Presini, Federico Zappaterra, Pier Paolo Giovannini, Lindomar Alberto Lerin

**Affiliations:** ^1^ Department of Chemical, Pharmaceutical and Agricultural Sciences University of Ferrara Ferrara Italy; ^2^ Department of Chemical Engineering and Food Engineering Federal University of Santa Catarina Florianópolis Brazil; ^3^ Department of Bioprocess Engineering and Biotechnology São Paulo State University Araraquara Brazil

**Keywords:** biocatalysis, enzymatic synthesis, geranyl benzoate, green chemistry, solvent‐free system

## Abstract

The demand for sustainable and natural alternatives in the flavor and fragrance industries has driven interest toward enzymatic synthetic routes. This study reports the enzymatic production of geranyl benzoate, a monoterpenoid ester with potential bioactivity and commercial applications, catalyzed by immobilized lipases using a solvent‐free system. A central composite design was employed to optimize the key process variables: molar ratio, temperature, and enzyme loading using Lipozyme 435 as the biocatalyst. Under optimal conditions (1:7 molar ratio, 80°C, 30% enzyme), a high geraniol conversion (72%) was achieved within 6 hr, as validated by a predictive model. The study also investigated the inhibitory effects of coproduced methanol, demonstrating that open‐reactor systems (enabling methanol evaporation) enhanced conversion (>99% in 48 h) compared to closed systems. Additionally, substrate‐exposure experiments highlighted methyl benzoate's inhibitory effect on Lipozyme 435, reducing its activity by 54%, whereas Lipura Flex showed greater resilience. Reusability tests indicated a decline in enzyme performance, with Lipozyme 435 retaining 22% activity after five cycles. Cell tests demonstrated no cytotoxicity and proliferative effect at high concentrations. These findings highlight the potential of solvent‐free enzymatic processes for sustainable production of geranyl benzoate and provide insights into operational challenges, including methanol management and biocatalyst stability.

## Graphical abstract

This study optimized solvent‐free geranyl benzoate production using Lipozyme 435, achieving 72% conversion in 6 hours. Open reactors improved yields (>99% in 48 hours) by removing inhibitorymethanol. Methyl benzoate reduced enzyme activity by 54%, though Lipura Flex showed better resistance. Human cell tests confirmed no cytotoxicity. These findings highlight the potential of solvent‐free enzymatic processes for sustainable fragrance production.

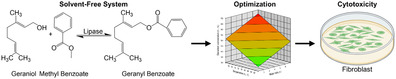

## 1 Introduction

The industrial chemistry sector is continually (and intensely) under pressure to become more sustainable and environmentally friendly, making green chemistry principles a guiding light for most advancements within the chemical industry. This shift is accompanied by a growing demand for increasingly complex products, often featuring multiple functionalities and well‐defined stereochemistry. In this context, the use of enzymes as industrial catalysts is gaining momentum [1, 2]. Enzymes are natural catalysts that can be considered either for their substrate specificity or for their catalytic promiscuity. They typically operate under mild temperature, pressure, and pH conditions, achieving high conversion and catalytic rates [3, 4]. Consequently, enzymatic synthesis is widely regarded as a forward‐looking and eco‐friendly alternative to traditional methods [5, 6]. Lipases, a subclass of esterases, play a crucial role in the digestion and processing of lipids. They catalyze reactions such as hydrolysis and esterification, acting at the interface between aqueous and nonaqueous phases [7]. Lipases are notable for their high specificity, efficiency in organic solvents, the absence of cofactors required, and nontoxicity. Microbial lipases are particularly favored due to their high productivity, stability, and ease of genetic manipulation. Host microorganisms can grow rapidly in economically viable media, and their production is scalable, making them ideal for industrial enzyme production [7, 8, 9, 10, 11].

Geraniol (Ger), known for its low human toxicity, is utilized as a natural pest control agent; however, its use in the food industry is limited due to its physicochemical properties. The esterification of Ger emerges as a solution, modifying its properties, such as polarity, volatility, and aroma, while expanding its applications [5, 12, 13]. Compared to Ger, its esters have a more pleasant floral aroma and are widely used in food, cosmetics, fragrances, and pharmaceutical products [14, 15, 16]. Traditionally, these esters are obtained through fermentation or extraction from natural sources; however, these methods are expensive and inefficient. Chemical synthesis is an industrial alternative but faces challenges such as harsh reaction conditions, unwanted byproducts, and environmental impacts associated with waste generation. Additionally, chemically produced esters are not considered natural [5]. According to the United States of America and European regulations, esters, such as the geranyl esters, produced by using biocatalysts, are classified as producing natural fragrances [5, 17, 18].

Geranyl benzoate [(2*E*)−3,7‐dimethyl‐2,6‐octadien‐1‐yl benzoate], a monoterpenoid ester present in the composition of some essential oils such as *Plumeria alba* L. (27.5%), *Plumeria rubra* L. (13.4%) [19], *Plumeria obtusa* L. (3.0%) [20], and *Cananga odorata* Hook. fil. et Thomson (3.1%) [21] is widely used as a flavoring agent and obtained the generally recognized as safe (GRAS) status by the Flavor and Extract Manufacturers Association (FEMA) [22] in 1965, and was approved for use in food by the Food and Agriculture Organization (FAO) and the World Health Organization (WHO) [23]. In a recent study, geranyl benzoate was subjected to docking analysis, showing a potential dual anticholinesterase activity (acetylcholinesterase inhibitors (AChEIs) and butyrylcholinesterase inhibitors (BChEIs)), demonstrating significant potential to alleviate symptoms and decrease the rate of cognitive deterioration caused by Alzheimer's disease [19].

Ger esters can be produced in different reactor configurations, operating in batch, fed‐batch, or continuous mode. Among these, batch‐stirred tank reactors (BSTR) are the most widely used for reactions with immobilized lipases due to their operational simplicity, ease of use, and relatively lower cost compared to other reactor types [11, 14, 16]. Studying process variables, such as substrate molar ratio, biocatalyst loading, agitation, and temperature, is essential because these factors significantly influence reaction equilibrium, viscosity, diffusivity, mass transfer, and enzyme kinetics, including enzyme inhibition. Thus, optimizing process variables is crucial to maximizing productivity and yield.

**SCHEME 1:**
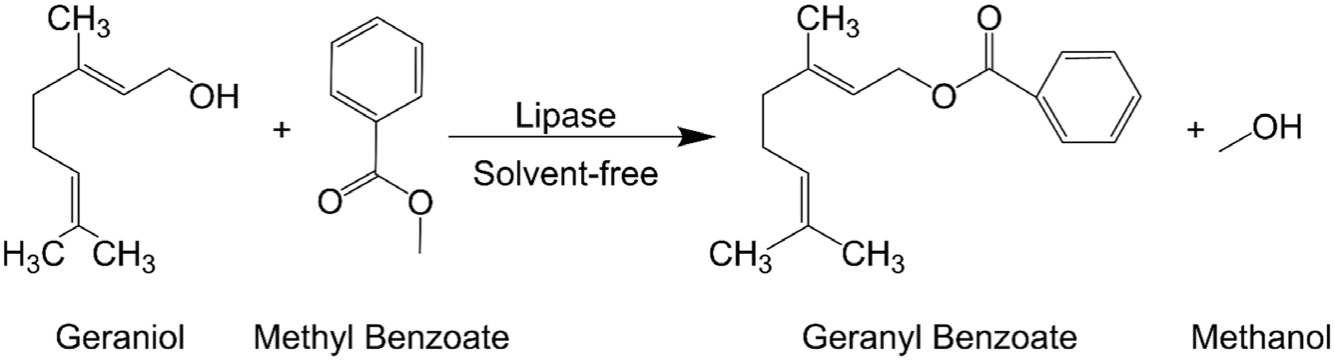
Production of geranyl benzoate by enzymatic transesterification of MeBenz and Ger.

Considering the above, this study explored the enzymatic production of geranyl benzoate (Scheme 1). The transesterification of methyl benzoate (MeBenz) with Ger, catalyzed by various lipases in a solvent‐free (SF) system using a BSTR, was optimized using experimental design (DoE), followed by evaluation of biocatalyst reusability and scale‐up. Subsequently, the purified geranyl benzoate was assessed for in vitro cytotoxicity.

## 2 Results and Discussion

### 2.1 Optimization of Geranyl Benzoate Production

This study aims to establish the optimal conditions for the lipase‐catalyzed transesterification of MeBenz with Ger. Given that multiple variables can significantly influence the reaction yield, a factorial experimental design (2^k^) was employed to (I) identify the key variables and (II) develop a predictive model to maximize geranyl benzoate production. This methodology applies statistical and mathematical principles to analyze multivariate problems, enabling the development of mathematical models, the identification of critical experimental factors, and the determination of key variables. For this purpose, a 2^3^ central composite design (CCD) was selected due to its advantages and well‐established use in bioprocess optimization. CCD requires fewer experimental runs while enabling a comprehensive analysis of variable effects and response surface behavior compared to a “one‐at‐a‐time” design [24]. The independent variables, molar ratio (Ger to MeBenz), temperature, and amount of immobilized lipase (expressed as a percentage of the total mass of the Ger), were selected for evaluation in the 2^3^ CCD. The experiments were conducted in a hermetically sealed reactor (closed reactor) with magnetic stirring at 500 rpm (Table 1).

**TABLE 1:** 2^3^ CCD matrix (coded and real (in brackets) values) and responses regarding experimental and predicted Ger conversion using Lipo 435 in an SF system.

Run	**Molar ratio** a	Temperature (°C)	**Enzyme amount, %** b	Ger conversion, %		
				Experimental	**Predicted** c	**RE, %** d
1	−1 (1:1)	−1 (60)	−1 (10)	26	25.7	1.2
2	1 (1:7)	−1 (60)	−1 (10)	40	41.0	−2.4
3	−1 (1:1)	1 (80)	−1 (10)	42	43.5	−3.5
4	1 (1:7)	1 (80)	−1 (10)	60	58.7	2.1
5	−1 (1:1)	−1 (60)	1 (30)	40	41.5	−3.7
6	1 (1:7)	−1 (60)	1 (30)	58	56.7	2.2
7	−1 (1:1)	1 (80)	1 (30)	61	59.2	2.9
8	1 (1:7)	1 (80)	1 (30)	72	74.5	−3.4
9	0 (1:4)	0 (70)	0 (20)	52	50.1	3.7
10	0 (1:4)	0 (70)	0 (20)	49	50.1	−2.2
11	0 (1:4)	0 (70)	0 (20)	51	50.1	1.8

a Molar ratio Ger to MeBenz.

b Enzyme amount as % weight concerning the mass of Ger.

c Calculated according to the response model (Table 2).

d $\text{Relative Error}\left(\mathrm{RE},\%\right)=\left(\frac{\text{Experimental conversion}-\text{Predicted conversion}}{\text{Experimental conversion}}\right)*100$.

Initially, the kinetics of reactions performed with different lipases (both immobilized and in liquid form) in the presence or absence of organic solvents were evaluated under the central‐point experimental conditions (Table 1, runs 9–11). These tests were conducted to determine which enzymes, systems, and reaction times would be used to optimize the production of geranyl benzoate. Four lipases immobilized on solid supports, namely Lipozyme 435 (Lipo 435), Lipura Flex (Lipura), Lipozyme RM IM, and Lipozyme TL IM, as well as three liquid lipase formulations (Novocor AD L, Novozym 51032 (Novo 51032), and Resinase HT), were evaluated in both SF systems and in the presence of solvents such as *tert*‐butanol (*t*‐BuOH), *tert*‐amyl alcohol (*t*‐AmOH), or 2‐methyltetrahydrofuran (2‐MeTHF).

**FIGURE 1:**
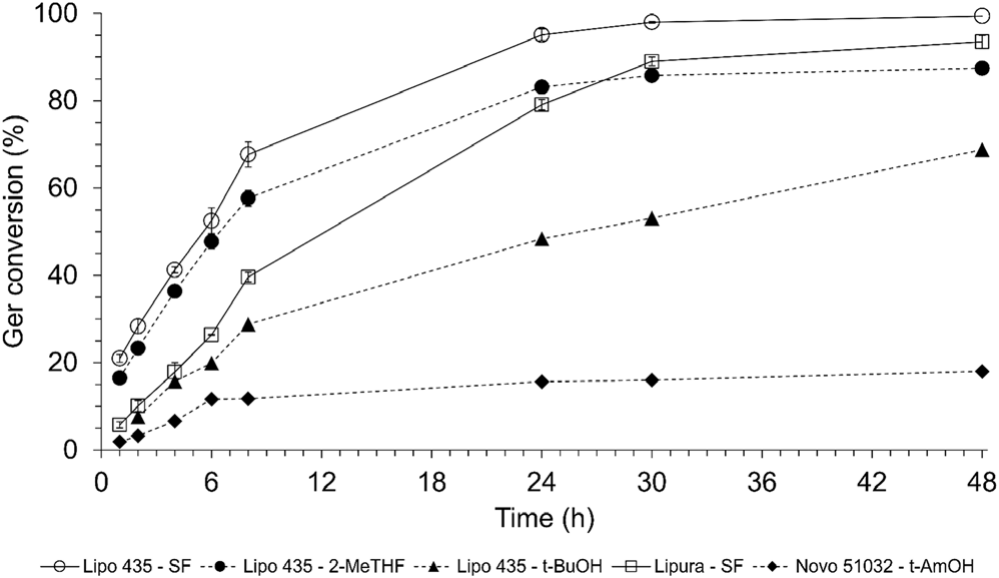
Kinetics of geranyl benzoate production catalyzed by different lipases in various reaction systems. Reaction conditions correspond to the 2^3^ CCD central point (Table 1, runs 9–10).

Figure 1 presents the main results obtained in this evaluation. The biocatalysts Lipo 435 and Lipura showed the best performance in the transesterification reactions. Both are lipase B from *Candida antarctica* but are immobilized on different supports. From the kinetics of Figure 1, the initial velocity (*V*_0_) was determined from the slope of the product formation curve in the first 6 hr of reaction. Lipo 435 exhibited *V*_0_ of 3.50, 3.00, and 1.83 μmol/min in the SF system, 2‐MeTHF, and *t*‐BuOH, respectively. In contrast, Lipura and Novo 51 032 exhibited their best *V*_0_ values of 2.50 μmol/min in the SF system and 1.00 μmol/min in the *t*‐AmOH system, respectively. The lower *V*_0_ of Lipura compared to Lipo 435 may be related to the smaller surface area, total pore volume, and average pore diameter of its support compared to those of Novozym 435 [25] (with Lipo 435 being its food‐grade version). These characteristics may reduce enzyme‐substrate contact, decreasing the formation of the enzyme–substrate complex, and, consequently, lowering *V*_0_.

Lipo 435 achieved higher conversions, 68% (SF) and 58% (2‐MeTHF), after 8 hr of reaction, while Lipura reached 40% (SF) conversion. However, the difference in conversion between the SF systems with Lipo 435 and Lipura decreased over time, from 28% at 8 hr to 16% at 24 hr, 9% at 30 hr, and finally to 6% at 48 hr. By the end of the 48‐h reaction, the conversion of Ger to the product was nearly identical in both cases, exceeding 94%. The system with *t*‐BuOH and Lipo 435 achieved a maximum conversion of 68%, whereas the system with *t*‐AmOH and Novo 51032 reached only 18% conversion after 48 hr of reaction (Figure 1). For the other lipases, conversions after 48 hr were below 5%, so these results were not included in the graphical representation.

The Lipo 435 performed best in the SF system. When comparing the activity of Lipo 435 and Lipura in the SF system, Lipo 435 showed a higher catalytic rate during the first hour. Over the next 5 h, however, the two biocatalysts exhibited similar activities, as indicated by the slope of the kinetic curves. At longer reaction times (>6 hr), Lipura became more active, enabling the enzyme‐catalyzed reaction to reach, after 48 h, a conversion comparable to that achieved with Lipo 435. Organic solvents had detrimental effects on all biocatalysts except Novo 51032; even with this biocatalyst, the results were unsatisfactory.

Based on the results of the lipase and reaction‐system screening for geranyl benzoate production, Lipo 435 in a SF system with a 6‐h reaction time was selected as the model for subsequent process optimization. This choice was made because SF systems provide higher productivity, generate less waste, and align with green chemistry principles, making them suitable for large‐scale biocatalytic synthesis. Table 1 presents the 2^3^ CCD matrix, including both coded and real values, along with the experimental and predicted responses (in terms of Ger conversion into product) from the empirical mathematical model and the corresponding relative errors (RE). Good results were obtained in the optimization, with conversions above 70% achieved within 6 hr of reaction. Another important observation from Table 1 is the high reproducibility of the tests, as evidenced by the repeated central point (runs 9–11), which resulted in a low pure error of 0.29% (calculated as (4.67/1591.38) × 100), approaching zero.

**FIGURE 2:**
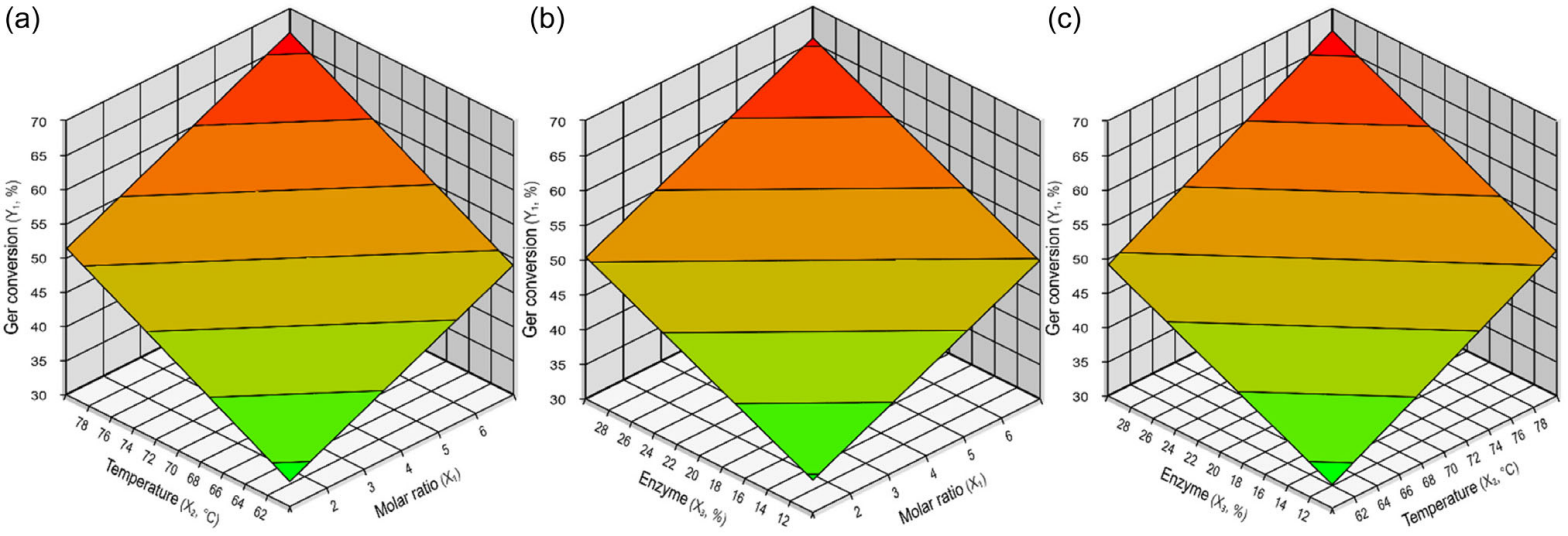
Response surface plots of Ger conversion to product as a function of molar ratio, temperature, and enzyme amount in an SF system using Lipo 435 as a biocatalyst were constructed from the equation shown in Table 2. For all tests, agitation was set to 500 rpm. In Figure (a), the enzyme load was 20%; in Figure (b), the reaction temperature was 70°C; and in Figure (c), the molar ratio was 1:4.

The effect of the independent variables was evaluated only for statistically significant responses at a 5% significance level (95% confidence level). Reparameterization (Table S1) was performed, and an empirical model describing the conversion (%) of Ger into geranyl benzoate as a function of molar ratio, temperature, and enzyme amount was obtained, as presented in Table 2. Analysis of variance (ANOVA; Table S2) was conducted to validate the empirical model (*p* < 0.05), and the results are summarized in Table 2. The empirical model was successfully validated, as evidenced by a coefficient of determination (R^2^) greater than 98% and calculated *F*‐values that were 36 times higher than the tabulated *F*‐value [24]. Consequently, the response surfaces for the conversion of Ger into the product could be generated, as illustrated in Figure 2.

**TABLE 2:** ANOVA to determine the percentage of explained variance (R^2^), the calculated *F*‐value (*F*_calc._), the tabulated *F*‐value (*F*_tab._), and the ratio *F*_calc._/*F*_tab._ for the Ger conversion response. Models are considered statistically significant when the *F*_calc._/*F*_tab._ ratio exceeds 1.

**Response model** a	**R** ^**2**^ **, %**	***F***_**calc.**_	***F***_**tab.**_	$\frac{F_{calc.}}{F_{tab.}}$
$Y_{1}=50.09+7.63MR+8.88T+7.88E$	98.54	157.78	4.34	36.34

a *y*_1_ is the % Ger conversion, *MR* is substrate molar ratio, *T* is temperature (°C), and *E* is enzyme amount (%).

The Ger conversion predicted by the model (Table 2) closely matched the experimental results, yielding low RE (Table 1). This demonstrates the model's excellent fit.

The analysis of the independent variables revealed a significant positive effect on Ger conversion, with higher molar ratios, temperatures, and enzyme amounts leading to increased conversions (Tables 1 and 2, Figure 2). The highest conversion (72%) was achieved in run 8, which combined the maximum values for all three variables: a 1:7 molar ratio, 80°C temperature, and 30% enzyme amount. This trend was further supported by other high conversion runs (Figure 2), including run 7 (61% conversion at the highest temperature and enzyme amount), run 6 (58% conversion with the highest molar ratio and enzyme loading), and run 4 (60% conversion under the highest molar ratio and temperature conditions). Figure 2, through the response surface, clearly demonstrates the positive effect of the independent variables on the conversion of Ger into the product.

The molar ratio of the substrates and the temperature play important roles in the reactions, as they influence reagent solubility and mass transfer. In SF reactions, the substrate excess, in this case MeBenz, acts as a solvent, shifting the reaction equilibrium. Temperature also influences methanol concentration, a coproduct of the reaction and a potential inhibitor of the lipase, as higher temperatures promote its evaporation, thereby reducing its concentration in the reaction medium, reducing the inhibitory effect [11, 14, 26, 27]. This observation is supported by the study of Venturi et al., who found that increasing temperature positively affects reactions in which the coproduced methanol was not removed. In contrast, no temperature effect was observed on the transesterification reaction when methanol was continuously sequestered [14]. Finally, the significant positive effect of the enzyme amount, particularly in SF systems, is attributed to the fact that high concentrations of substrates, products, and coproducts can result in inhibitory effects, which are more pronounced when lower enzyme amounts are used [14, 26, 27, 28]. The discussed effects are clearly illustrated in Figure 2, where low temperatures, lipase amounts, and molar ratios resulted in the lowest Ger conversions. As these parameters increased, conversion rates increased.

To validate the empirical model, the reactions were performed in duplicate under the conditions specified in Table 3, with a reaction time of 6 hr. Validation was based on calculating the RE between the experimental data and the predicted model values. The results demonstrate that the model aligns well with the experimental data, with REs below 10% in all cases, thereby confirming the empirical model's validity. In Table 3, run 3 recorded the highest conversion (71.0%) and an RE of 5.2%, whereas run 7, with the lowest conversion (35.5%), showed a RE of 4.9%. Runs 1 and 5 exhibited conversions of 54.0% (RE of 9.3%) and 62.4% (RE of 3.22%), respectively.

**TABLE 3:** Experimental conditions used to validate the empirical model (Table 2), with experimental and predicted conversion, and *V*_0_.

Biocatalyst	**Molar ratio** a	Temperature (°C)	**Enzyme amount, %** b	**Ger conversion, %** d		***V***_**0**_ **,** **μmol/min**
**Lipo 435** **Lipura**				**Experimental ± SD** e	**Predicted** c **± SD** e	
1*	1:7	80	10	53.69 ± 0.10	58.70 ± 9.32	4.00
2				33.29 ± 0.42	—	2.00
3*	1:7	80	30	70.77 ± 1.47	74.50 ± 5.27	5.16
4				50.58 ± 1.41	—	2.83
5	1:7	73	20	62.40 ± 1.48	60.39 ± 0.87	3.16
6				43.46 ± 0.82	—	2.66
7	1:2	70	10	35.50 ± 4.82	37.26 ± 0.95	2.50
8				33.66 ± 0.50	—	1.83

a Molar ratio Ger to MeBenz.

b Enzyme amount as % weight concerning the mass of Ger.

c Calculated according to the response model (Table 2).

d Ger conversion in 6 hr.

e Standard deviation.

* Optimized condition in the CCD (runs 4 and 8, Table 1).

**FIGURE 3:**
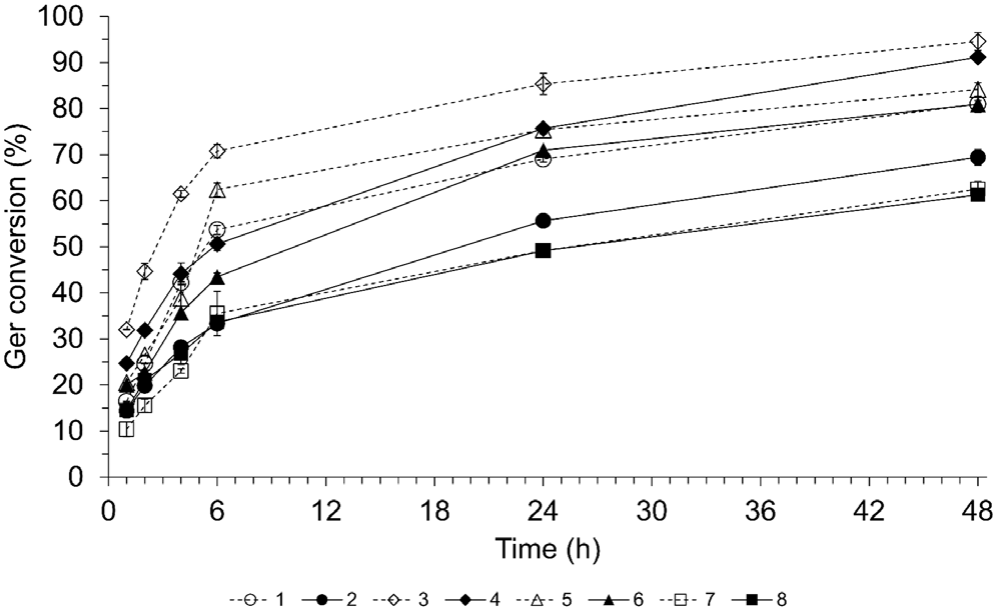
Validation of the empirical model through comparison of model predictions (Table 2) with 6‐h experimental data, along with kinetic evaluation to characterize the long‐term behavior of geranyl benzoate production in the SF system under the conditions detailed in Table 3.

Concurrently with the validation, the reaction kinetics were monitored to evaluate the effect of these conditions (Table 3) on Ger conversion (Figure 3) using Lipo 435 and Lipura as biocatalysts. As expected, increasing the amount of enzyme increases the *V*_0_. Lipo 435 exhibited the highest *V*_0_, with values of 2.50–5.16 μmol/min for reactions containing 30% to 10% enzyme. In contrast, Lipura showed lower *V*_0_ values of 1.83–2.83 μmol/min at the same enzyme concentration. In these experiments, it is worth noting that the enzyme amount significantly influences the reaction rate, particularly during the first 5 hr. However, during the subsequent reaction period, the kinetic curves for reactions catalyzed by different amounts of the same enzyme exhibit similar trends. These results are clearly illustrated in Figure 3. In reactions conducted with 30% enzyme, Lipura displayed slower kinetics compared to Lipo 435, as evidenced by its lower *V*_0_. The difference in Ger conversion after 6 hr was ≈20%; however, after 48 hr, this difference diminished to less than 4%, with both enzymes achieving conversions of around 93%. The reaction kinetics were slower with the 10% enzyme than with the 30% enzyme. The maximum conversions reached after 48 hr were 80% for Lipo 435 and 69% for Lipura. These results align with those shown in Figure 1, where Lipura exhibited slightly higher activity than Lipo 435 at longer reaction times. This allowed the reaction with 30% Lipura to achieve a conversion comparable to that of 30% Lipo 435 after 48 hr. However, this difference is less pronounced at a lower enzyme concentration (10%), likely because the inhibitory effects of coproduced methanol become more significant with reduced enzyme amounts. From these results, it is evident that as lipase levels decreased, the effect of methanol became more pronounced, as previously discussed. Another relevant finding is that the lowest values of *V*_0_ and conversion (runs 7 and 8, Table 3) were observed at the lowest levels (−1) of the CCD. This result confirms the positive effect of the investigated variables. Under these conditions, the reaction kinetics were significantly slower for both enzymes, reaching a maximum conversion of only 61% after 48 hr, a value at least 20% lower than that of the other tested conditions.

Few studies have explored the use of Lipura in synthesis processes, and only one has evaluated its reaction kinetics [25, 29, 30, 31]. In contrast to our findings, Venturi et al. reported that Lipura was more efficient in transesterification reactions between methyl acetoacetate and ursodeoxycholic acid [29]. The reaction kinetics catalyzed by Lipura varied significantly across the substrates studied by Remonatto et al. [31]. While the conversion to geranyl acetate reached 99% within 1 hr, indicating rapid kinetics, the conversion to geranyl cinnamate took 24 hr to reach 100%, demonstrating a much slower reaction rate.

### 2.2 Effects of Substrates and Coproduced Methanol

Enzymatic (trans)esterification is a thermodynamically controlled process in which lipases can be inhibited or inactivated by substrates, products, or heat. Additionally, while a certain amount of water is essential for catalytic activity, excess water favors hydrolysis, reducing the (trans)esterification yield [26]. High methanol concentrations negatively impact enzymatic activity through hydrophobic interactions and hydrogen bonds, leading to denaturation or structural changes. Methanol‐induced lipase inactivation primarily occurs through conformational changes that lead to enzymatic aggregation and detachment from the solid support. Additionally, evidence suggests that other factors, including water activity, competitive inhibition, and effects on local conformational dynamics, influence this process [14, 32].

With this in mind, the effect of methanol coproduced during the transesterification reaction of MeBenz was evaluated. For this purpose, kinetic studies were conducted under conditions previously optimized and validated (runs 1–8) and with reduced enzyme amount (runs 9–12); the conditions are presented in Table 4. In the runs carried out in the hermetically sealed reactor (closed reactor), lower *V*_0_ were observed (2.50–3.33 μmol/min for Lipo 435 and 1.83–2.50 μmol/min for Lipura) compared to reactions in the open reactor (3.16–10.66 μmol/min for Lipo 435 and 3.33–9.16 μmol/min for Lipura). An exception was the condition at 80°C with Lipo 435, where the *V*_0_ in the closed reactor was slightly higher (runs 1 and 3, Table 4).

**TABLE 4:** Experimental conditions used to evaluate the effect of substrates and coproduced methanol, with experimental conversion, and *V*_0_.

Biocatalyst	**Molar ratio** a	Temperature, °C	**Enzyme amount, %** b	**Ger conversion, %** c **± SD** d	***V***_**0**_ **, μmol/min**
**Lipo 435** **Lipura**					
Closed 1*	1:7	80	10	69.01 ± 0.66	4.00
Closed 2	1:7	80	10	55.62 ± 0.10	2.00
Open 3	1:7	80	10	99.13 ± 0.21	3.16
Open 4	1.7	80	10	98.65 ± 0.28	3.33
Closed 5**	1:2	70	10	49.17 ± 1.40	2.50
Closed 6	1:2	70	10	49.11 ± 0.39	1.83
Open 7	1:2	70	10	97.63 ± 0.12	10.66
Open 8	1:2	70	10	98.16 ± 0.47	9.16
Closed 9	1:2	70	5	45.25 ± 3.44	3.33
Closed 10	1:2	70	5	35.37 ± 0.65	2.50
Open 11	1:2	70	5	91.75 ± 0.30	5.50
Open 12	1:2	70	5	89.23 ± 2.02	7.16

a Molar ratio Ger to MeBenz.

b Enzyme amount as % weight concerning the mass of Ger.

c Ger conversion in 24 h.

d Standard deviation.

* CCD optimized and validated conditions (Tables 1 and 3).

** CCD validated conditions in Table 3.

**FIGURE 4:**
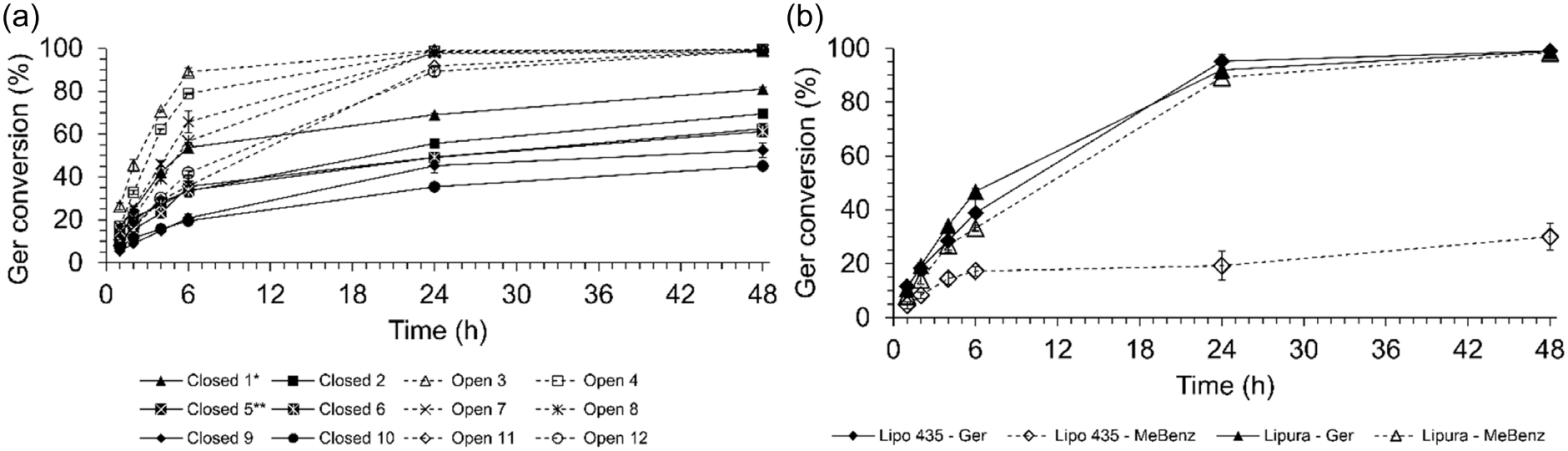
Impact of (a) coproduced methanol, experimental condition of Table 4, and (b) substrates, experimental condition of Open 11 and 12 of Table 4, on transesterification efficiency. *CCD optimized and validated conditions (Tables 1 and 3). **CCD validated conditions in Table 3.

In the closed reactor, the conversions for both lipases did not exceed 69% at 24 hr or 80% at 48 hr (Figure 4a), with the highest values being observed at higher temperatures and higher molar ratios. Furthermore, under these conditions, the methanol concentration in the reaction medium, calculated based on the conversion values, exceeded 1.5% at 6 hr, 1.6% at 24 hr, and 2.0% at 48 hr for both enzymes.

In contrast, in the open reactor experiments, conversions exceeded 90% and 99% for both lipases at 24 hr and 48 hr, respectively (Figure 4a). The reaction kinetics were significantly faster in this system, resulting in a higher *V*_0_ and, consequently, a higher conversion of Ger to geranyl benzoate. Even in reactions with the lowest enzyme amount (5%), which showed a slower initial reaction rate, a conversion equivalent to that in the other reaction conditions was achieved after 48 hr. This effect is attributed to the continuous removal of methanol from the reaction medium by evaporation, as the reaction temperature exceeded its boiling point (64.7°C).

According to Venturi et al., harsher process conditions were required for the enzymatic transesterification of methyl acetoacetate with Ger in closed systems without methanol removal. However, when methanol was captured using molecular sieves, the effect of temperature became negligible, allowing high conversions to be achieved under mild process conditions [14]. A recent study demonstrated that methanol concentrations exceeding 0.2% (wt%) are unsuitable for achieving fatty acid contents above 95% in the enzymatic hydrolysis of fatty acid methyl esters [33]. Another study found that methanol concentrations above 1% inhibit *C. antarctica* lipase B [34]. Therefore, developing effective and efficient strategies for removing coproduced alcohols in enzyme‐catalyzed processes is crucial.

A stoichiometric excess of a reagent can alter the physical properties of the reaction medium, including polarity, viscosity, solubility, and pH, particularly in SF systems, where reactants and products constitute the medium. Additionally, it may denature, inactivate, or inhibit the enzyme if the reagent adversely affects the biocatalyst [26]. Therefore, another aspect that needs to be explored is the effect of lipase exposure to different substrates. To investigate this, experiments were conducted under the previously described reaction conditions in an open reactor to eliminate the effect of methanol (runs 11 and 12, Table 4). The lipases were incubated at the reaction temperature and agitation for 2 hr, using the precise amount of the corresponding substrate and enzyme intended for the reaction (Figure 4b). The reaction was initiated by adding the missing substrate.

Exposing both lipases to Ger did not change their *V*_0_. For Lipo 435, *V*_0_ was 5.83 μmol/min, and for Lipura, 7.67 μmol/min. The reactions yielded over 90% geranyl benzoate within 24 hr, reaching near‐complete conversion (99%) in 48 hr. When exposed to MeBenz, Lipura exhibited a ≈30% decrease in *V*_0_ (5.33 μmol/min) but maintained kinetics similar to those observed with Ger, achieving > 98% conversion in 48 hr. In contrast, Lipo 435 showed a reduction of more than 54% in V_0_ (2.66 μmol/min) and a decrease of more than 70% in conversion, reaching only 30% after 48 hr, which is significantly lower than its performance with Ger (Figure 4b). Ceni et al. found that high concentrations of MeBenz inhibited Novozym 435 lipase, reducing the reaction rate [35]. Shinde et al. also observed inhibition by MeBenz. However, they ruled out a ping–pong bi–bi mechanism and instead proposed a complex ternary mechanism involving inhibition by n‐hexanol [36].

The inactivation of Lipo 435 may be related to the support's moderate hydrophobicity, which facilitates the retention of hydrophilic substrates such as MeBenz (log *p* = 2.2). This retention could cause the enzyme to lose the essential water layer required for flexibility, leaving the protein more rigid and preventing the structural changes necessary for catalysis. However, this correlation is limited, as the effects are inconsistent across lipases from different sources or those subjected to different immobilization protocols [26, 28, 37, 38]. As observed in Figure 4b, lipase B from *C. antarctica* exhibited very different behaviors on the two supports: Lipura did not show a decrease in Ger conversion when in contact with MeBenz, whereas Lipo 435 showed a significant reduction (70%). However, the inhibitory effect of MeBenz was not observed in the reaction mixtures. In all proportions tested in this study, Lipo 435 showed no reduction in its synthetic activity.

### 2.3 Reusability of Immobilized Lipases

The economic viability of biocatalytic routes depends on high reagent conversion, product quality, and enzyme reusability. In ester synthesis, studies on lipases typically assess reuse using a standard approach that involves sequential reactions under predefined conditions, along with washing and drying protocols between batches. However, this strategy may compromise enzyme stability and hinder industrial feasibility due to additional operational steps, increased material and equipment costs, and unproductive downtime [37, 39]. Here, the reusability of Lipo 435 and Lipura was investigated without organic solvent washing to assess their stability over successive 24‐h reaction cycles. The reactions were conducted under the following conditions: molar ratio of 1:2 (Ger to MeBenz), 5% enzyme loading (relative to Ger mass), 70°C, and 500 rpm magnetic stirring.

**FIGURE 5:**
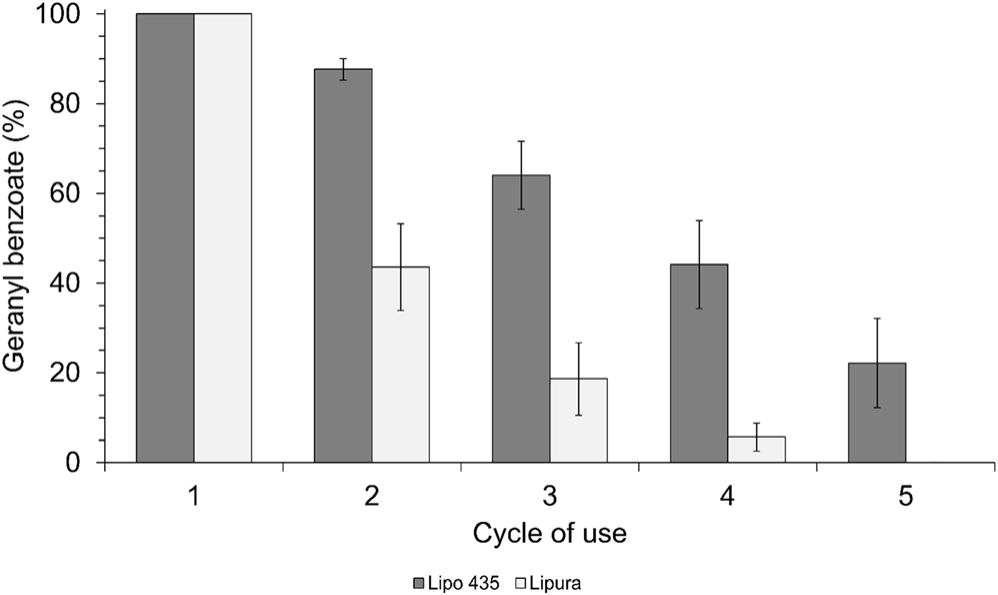
Reusability of lipases in the conversion of Ger to produce geranyl benzoate in SF systems. Reaction conditions: 1:2 molar ratio, 5% enzyme amount, a temperature of 70°C, and 500 rpm.

Both lipases exhibited a progressive decline in catalytic activity starting from the second cycle of use (Figure 5). For Lipo 435, the reduction in conversion was modest (12%) in the second cycle, but subsequent cycles showed more significant losses, reaching 78% by the fifth cycle. In contrast, Lipura experienced a more significant decline, with a loss exceeding 57% in the second cycle and reaching 96% by the fourth. By the fifth cycle, Lipura showed no detectable Ger conversion (Figure 5). These results contradict literature reports highlighting Novozym 435's robust stability in Ger ester synthesis, even in SF systems [14]. No prior studies on Lipura's reusability were identified. The observed conversion losses in both lipases are likely due to enzymatic deactivation, driven by multiple factors: prolonged substrate/product exposure, dehydration of the enzyme's microenvironment, thermal denaturation, mass transfer limitations, enzymatic leaching, and intraparticle diffusion barriers. Additionally, mechanical stress from magnetic stirring may compromise the support's integrity, disrupt enzyme‐support binding, or cause pore blockage, thereby restricting substrate access to the active site [14, 37, 39, 40].

In this study, no inhibitory effects from the reaction mixture or methanol in an open reactor were observed, nor was any effect of temperature, since both lipases showed good stability at elevated temperatures. No visible modifications or destruction of the enzyme support were observed either. The decrease in geranyl benzoate production is likely due to enzyme leaching from the support. Due to the weak adsorption forces, the leakage of proteins thus bound to the carrier can be easily triggered by slight changes in temperature, pH, and ionic strength, or even by the presence of substrates and solvents [40].

### 2.4 Process Scalability

**FIGURE 6:**
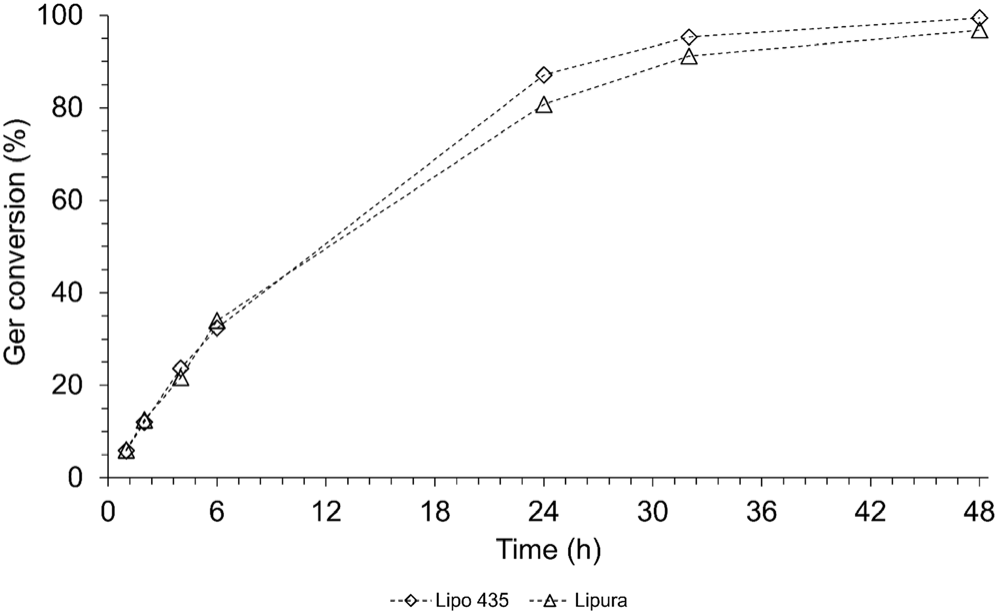
Scalability of geranyl benzoate production in SF systems. Reaction conditions: 1:2 molar ratio, 5% enzyme amount, a temperature of 70°C, and mechanical agitation of 500 rpm.

To evaluate the scalability of the method, a scaled‐up synthesis (100‐fold increase, final reaction volume of 250 mL) of geranyl benzoate was performed using the maximized conditions (runs 11 and 12, Table 4) with mechanical stirring at 500 rpm. The results, presented in Figure 6, demonstrate that both the *V*_0_ (*V*_0_ of 34.5 µmol/min for Lipo 435 and 35.3 µmol/min for Lipura) and the final conversion (>97% in 48 hr for both lipases) remained consistent after scale‐up, closely resembling the data obtained at the laboratory scale (2.5 mL). Therefore, it is concluded that the approach developed in this study for the synthesis of geranyl benzoate is not only applicable at the laboratory scale but also shows promising potential for industrial implementation.

In industrial reactors, the removal of coproduced methanol must be optimized, as at high volumes, temperature control alone may be insufficient to eliminate it from the reaction mixture adequately. The residual presence of methanol can compromise the reaction equilibrium and product quality. Therefore, the implementation of vacuum or membrane systems is proposed for the efficient removal of methanol without significant losses of substrates and product. Although membrane technology is promising, its initial investment cost is higher than that of vacuum distillation. A vacuum system operating between 100 and 200 mBar, for example, reduces the boiling point of methanol to 10°C–24°C, allowing easy distillation from the mixture. Additionally, this system enables the recovery and reuse of methanol in other processes by condensing it at suitable temperatures.

### 2.5 Cytotoxicity Assay

Although geranyl benzoate has GRAS status and is approved for food use by the FAO and WHO, with safety assessments indicating an acceptable risk at known use levels [22, 23]. Nevertheless, published data on its cytotoxicity and genotoxicity are scarce [41]. In contrast, studies on structural analogs report varying effects; for instance, geranyl acetate reduced cell viability by 30%, whereas geranyl cinnamate was nontoxic to L929 fibroblast cells [31]. At the same time, Ger and geranylated derivatives demonstrated cytotoxic activity in several cell lines [42, 43]. Given its proposed novel biocatalytic production and potential applications in fragrances, food, and cosmetics, it is imperative to evaluate the cytotoxicity of geranyl benzoate to confirm its biocompatibility and safety.

**FIGURE 7:**
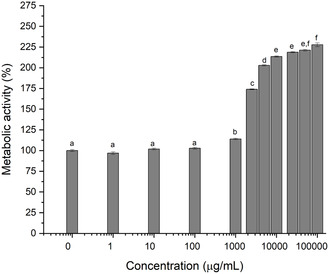
Metabolic activity of the fibroblast lineage L929 after exposure to solutions of geranyl benzoate.

Geranyl benzoate, produced enzymatically, exhibited no cytotoxicity toward L929 cells at concentrations up to 1000 µg/mL, as determined by a metabolic activity assay (Figure 7). Interestingly, at the highest concentration (1000–100,000 µg/mL), a significant increase in metabolic activity of up to 125% was observed, potentially indicating enhanced cell proliferation. This stimulatory effect is a positive indicator of biocompatibility, suggesting that geranyl benzoate may possess bioactive properties conducive to applications such as tissue regeneration. Consequently, these findings not only support the compound's safety but also guarantee further investigation into its bioactivity. However, further studies should be conducted to confirm other biological activities, such as wound healing, cell proliferation, antioxidant, and antimicrobial effects.

## 3 Conclusion

This study demonstrated the feasibility and efficiency of enzymatic transesterification for producing geranyl benzoate in an SF system using immobilized lipases, particularly Lipo 435 and Lipura. Optimization using a 2^3^ CCD revealed that higher molar ratios (1:7, Ger to MeBenz), elevated temperatures (80°C), and increased enzyme amount (30%) significantly enhanced reaction efficiency, achieving up to 72% conversion in 6 hr with Lipo 435. The predictive model developed showed an excellent correlation with experimental data, indicating strong statistical reliability. Kinetic studies revealed that methanol accumulation significantly inhibits enzymatic activity, and its removal via evaporation in open reactors markedly enhances conversion (>99%). This resulted in maximized conditions for producing geranyl benzoate with a molar ratio of 1:2, 5% lipase amount, and a temperature of 70°C. The process scalability assessment concluded that it is scalable, maintaining high productivity (97%) over 48 hr. However, challenges were observed, including gradual enzyme leaching during reuse, highlighting the need for further research on biocatalyst stabilization strategies. No cytotoxicity of the geranyl benzoate was observed at concentrations up to 1000 µg/mL. Concentrations above this still caused clearance proliferation of up to 125%. These results highlight the advantages of enzymatic catalysis as a sustainable and scalable alternative to conventional chemical methods, aligning with the principles of green chemistry and natural product synthesis.

## 4 Experimental Section

### 4.1 Chemicals and Enzymes

Commercial lipases from *C. antarctica* (CalB) immobilized on macroporous acrylic resin (Lipozyme 435 – Lipo 435), on polymeric acrylic resin (Lipura Flex – Lipura), and CalA in liquid form (Novocor AD L); from *Rhizomucor miehei* immobilized on macroporous anion exchange resin (Lipozyme RM IM); from *Thermomyces lanuginosus* immobilized on a noncompressible silica gel carrier (Lipozyme TL IM); and from *Humicola insolens* (Novozym 51032–Novo 51032) and *Aspergillus oryzae* (Resinase HT), both in liquid form, were kindly donated by Novozymes A/S. Methyl benzoate (MeBenz), geraniol (Ger), *tert*‐butanol (*t*‐BuOH), *tert*‐amyl alcohol (*t*‐AmOH), 2‐methyltetrahydrofuran (2‐MeTHF), and acetic acid were purchased from Sigma–Aldrich. Cyclohexane and ethyl acetate were purchased from Carlo Erba.

### 4.2 Optimization of Geranyl Benzoate Production

The reactions were performed in a hermetically sealed 5 mL glass reactor with a concave bottom, with an approximate reaction volume of 2.5 mL. Stirring was performed using a magnetic stir bar, and the temperature was controlled by immersing the reactor in a temperature‐regulated oil bath equipped with stirring control.

The optimization of geranyl benzoate production in a SF system was performed using a 2^3^ CCD with triplicate central points. The process variables studied included a substrate molar ratio of 1:1 to 1:7 (Ger to MeBenz), a temperature range of 60°C–80°C, and an enzyme amount of 10% to 30% (expressed as a percentage of the mass of Ger). Agitation was maintained at 500 rpm throughout all experiments. The combined process variables from all runs are presented in Table 1. The choice of lipase, time, and reaction system for the CCD was determined by kinetic evaluation under central‐point conditions (runs 9–10, Table 1). The empirical mathematical model was validated under the conditions listed in Table 3 by monitoring reaction kinetics for 48 hr.

The Protimiza Experimental Design online tool (available at http://experimentaldesign.protimiza.com.br/) was utilized to support the design and statistical evaluation of the CCD. A confidence level of 95% (*p* ≤ 0.05) was applied.

### 4.3 Effects of Substrates and Coproduced Methanol

To evaluate the effect of methanol, the reactions were conducted in 5 mL reactors, hermetically sealed or open, under previously optimized and validated conditions, with a decrease in enzyme amount (Table 4) to maximize geranyl benzoate production in an SF system.

The reaction conditions for evaluating the effect of the substrates correspond to runs 11 and 12 in Table 4 (molar ratio of 1:2, 70°C, 5% enzyme amount, and 500 rpm) for the open reactor. The lipases were preincubated at the reaction temperature for 2 hr, using the precise amount of the corresponding substrate and enzyme intended for the reaction. The reaction was then initiated by adding the second substrate.

For both evaluations, reaction kinetics were monitored for 48 hr to observe the reaction progress under the different conditions. At each kinetic time, 10 μL of the reaction mixture was collected for subsequent quantification by gas chromatography. All reactions were performed in duplicate.

### 4.4 Lipase Reusability

In the lipase reuse, the reactions were carried out under maximized conditions in an open reactor (molar ratio of 1:2, 70°C, 5% enzyme amount, and 500 rpm). At the end of each cycle of use (24 hr), the reaction medium was separated from the biocatalyst by filtration through filter paper. The lipase was then reused for a new reaction without any additional treatment. At the end of each reaction cycle, the Ger conversion was quantified by gas chromatography. All reactions were performed in duplicate.

### 4.5 Scale‐Up Reaction

A 100‐fold scale‐up reaction was performed in an open 500 mL glass reactor with a concave bottom, with an approximate reaction volume of 250 mL under the maximized conditions: substrate molar ratio of Ger to MeBenz of 1:2, enzyme (Lipo 435 or Lipura) amount of 5% (expressed as a percentage of the mass of Ger), reaction temperature of 70°C. Mechanical agitation (500 rpm) was performed using a rod with a propeller, and the temperature was controlled by immersing the reactor in a temperature‐regulated oil bath. At each kinetic time, 10 μL of the reaction mixture was collected for subsequent quantification by gas chromatography. All reactions were performed in duplicate.

### 4.6 Geranyl Benzoate Quantification

The collected samples were diluted in ethyl acetate (1:100 v/v) before gas chromatographic analysis. Quantitation was performed on a PerkinElmer GC equipped with a flame ionization detector (FID) and a Zebron ZB‐5 column from Phenomenex (60 m × 0.25 mm × 0.10 μm). The oven temperature program began at 80°C (held for 2 min), followed by a ramp of 10°C/min to 300°C (held for 1 min). The injector temperature was set at 175°C, and the detector temperature was maintained at 320°C. The injection volume was 1 μL in split mode (ratio 1:50), using H_2_ as the carrier gas. The conversion of Ger to the product was performed according to the method described by Venturi et al. [14]

### 4.7 Geranyl Benzoate Purification and Spectroscopic Characterization

The reaction mixture was filtered to separate the biocatalyst, and the liquid phase was evaporated under reduced pressure (70°C and 100 Pa) to remove excess MeBenz. Subsequently, the residue was purified by column chromatography on silica gel (60 Å, 70–230 mesh, particle size 63–200 μm; Sigma–Aldrich) using a cyclohexane/ethyl acetate (7:1 v/v) eluent. The purified product was analyzed by ^1^H and ^13^C NMR spectroscopy at room temperature using a 400 MHz spectrometer with CDCl_3_ as the solvent (Figures S4 and S5).

Geranyl benzoate – ^1^H NMR (400 MHz, CDCl_3_) δ 8.07–8.00 (m, 2H), 7.57–7.50 (m, 1H), 7.39–7.46 (m, 2H), 5.50–5.43 (m, 1H), 5.13–5.05 (m, 1H), 4.84 (d, *J* = 7.1 Hz, 2H), 2.17–2.03 (m, 4H), 1.77 (s, 3H), 1.67 (s, 3H), 1.60 (s, 3H). ^13^C NMR (101 MHz, CDCl_3_) δ 166.65, 142.33, 132.75, 131.83, 130.51, 129.57, 128.26, 123.72, 118.37, 61.86, 39.53, 26.29, 25.65, 17.68, 16.55.

### 4.8 Cytotoxicity Assay

Cytotoxicity assays were performed according to the method described by ISO 10 993–5 (2009) [44, 45], where human cell lines, normal fibroblast cells (L929), were plated in 96‐well plates at a density of 1×10^4^ cells/well. Stock solutions were prepared in medium, followed by successive dilutions in complete medium to concentrations of 1–100,000 μg/mL, after 24 hr for complete adhesion. The compound was added, and the metabolic activity was measured using the MTS solution [46] after 24 hr of contact between the geranyl benzoate and the cells.

The dataset was evaluated by a one‐way analysis of variance (ANOVA). Using the *t*‐test or Tukey test, significant differences were determined at the 5% significance level (*p* < 0.05).

## Supporting Information

Additional supporting information can be found online in the Supporting Information section. **Supporting** **Fig.** **S1:** Pareto chart with the significant effects of the independent variables studied on the enzymatic synthesis of geranyl benzoate in a solvent‐free system (*p*<0.05). X_1_ represents the molar ratio, X_2_ represents the temperature, and X_3_ represents the enzyme amount. The experimental data and conditions are shown in Table 1. **Supporting Fig. S2:** Experimental versus predicted geraniol conversion for the enzymatic synthesis of geranyl benzoate. **Supporting Fig. S3:** Typical gas chromatogram of geranyl benzoate after removal of the excess of methyl benzoate by vacuum distillation (rotary evaporator, 70 °C, 200 rpm, 10 mBar). Retention time: geraniol 9.62 min, and geranyl benzoate 17.05 min. **Supporting Fig. S4:** ^1^H‐NMR spectra of geranyl benzoate. **Supporting Fig. S5:** ^13^C‐NMR spectra of geranyl benzoate. **Supporting Table S1:** Reparametrized regression coefficients of the central composite design (23‐factorial full design with triplicate center point) to estimate the effects of process variables on the enzymatic synthesis of geranyl benzoate. **Supporting Table S2:** Analysis of variance (ANOVA) of the estimated model for optimizing the enzymatic synthesis of geranyl benzoate.

## Conflicts of Interest

The authors declare no conflicts of interest.

## Supporting information

Supplementary Material

## Data Availability

The data that support the findings of this study are available from the corresponding author upon reasonable request.
