# Supplementary material for: Development of a Sustainable Biocatalytic Process for Geranyl Benzoate Production Using Immobilized Lipase
Source: ChemistryOpen. 2026 Feb 12;15(4):e202500476. doi: 10.1002/open.202500476 (PMC13052170; doi:10.1002/open.202500476)
Supplement: Supplementary file 1 — Supplementary Material [file OPEN-15-e202500476-s001.pdf]

SUPPORTING INFORMATION

Development of a Sustainable Biocatalytic Process for Geranyl Benzoate Production Using Immobilized Lipase

Domenico Meola,[a] Chaimae Chaibi,[a] Simona Aprile,[a] Karina Cesca,[b] Debora de Oliveira,[b] Ariela Veloso de Paula,[c] Francesco Presini,*[a] Federico Zappaterra,[a] Pier Paolo Giovannini,[a] and Lindomar Alberto Lerin*[a]

[a] D. Meola, C. Chaibi, S. Aprile, F. Presini, F. Zappaterra, P. P. Giovannini, L. A. Lerin
Department of Chemical, Pharmaceutical and Agricultural Sciences
University of Ferrara
Via Luigi Borsari, n. 46, 44121, Ferrara – IT
E-mail: prsfnc@unife.it; lrnldm@unife.it

[b] K. Cesca
Department of Chemical Engineering and Food Engineering
Federal University of Santa Catarina
Campus Reitor João David Ferreira Lima, 88040–900, Florianópolis – BR

[c] A. V. de Paula
Department of Bioprocess Engineering and Biotechnology
São Paulo State University
Rodovia Araraquara Jaú, Km 01 - s/n - Campos Ville, 14800-903, Araraquara, Brazil – BR

Contents

1. Supporting Tables

Table S1. Reparametrized regression coefficients of the central composite design (2^3-factorial full design with triplicate center point) to estimate the effects of process variables on the enzymatic synthesis of geranyl benzoate.
Table S2. Analysis of variance (ANOVA) of the estimated model for optimizing the enzymatic synthesis of geranyl benzoate.

2. Supporting Figures

Figure S1. Pareto chart with the significant effects of the independent variables studied on the enzymatic synthesis of geranyl benzoate in a solvent-free system (p<0.05). X_1 represents the molar ratio, X_2 represents the temperature, and X_3 represents the enzyme amount. The experimental data and conditions are presented in Table 1.
Figure S2. Experimental versus predicted geraniol conversion for the enzymatic synthesis of geranyl benzoate.
Figure S3. Typical gas chromatogram of geranyl benzoate after removal of the excess of methyl benzoate by vacuum distillation (rotary evaporator, 70 °C, 200 rpm, 10 mBar). Retention time: geraniol 9.62 min, and geranyl benzoate 17.05 min.
Figure S4. ^{1}H-NMR spectra of geranyl benzoate.
Figure S5. ^{13}C-NMR spectra of geranyl benzoate.

Table S1. Reparametrized regression coefficients of the central composite design (2^3-factorial full design with triplicate center point) to estimate the effects of process variables on the enzymatic synthesis of geranyl benzoate.

Factor	Coefficients	Standard Error	Calculated *t*	*p*-value
Mean	50.090	0.552	90.605	$5.25E^{-12}$
Molar ratio	7.625	0.648	11.762	$7.272E^{-6}$
Temperature	8.875	0.648	13.690	$2.614E^{-6}$
Enzyme amount	7.875	0.648	12.147	$5.856E^{-6}$

Table S2. Analysis of variance (ANOVA) of the estimated model for optimizing the enzymatic synthesis of geranyl benzoate.

Variation source	Sum of squares	Degrees of freedom	Mean square	F_{calc}	*p*-value
Regression	1,591.38	3	530.46	157.78	$8.65E^{-7}$
Residuals	23.53	7	3.36		
Lack of Fit	18.87	5	3.77	1.62	$4.25E^{-1}$
Pure Error	4.67	2	2.33		
Total	1,614.91	10			

Regression coefficient: $R^2 = 0.98$; $F_{0.95;\,3;\,7} = 4.34$

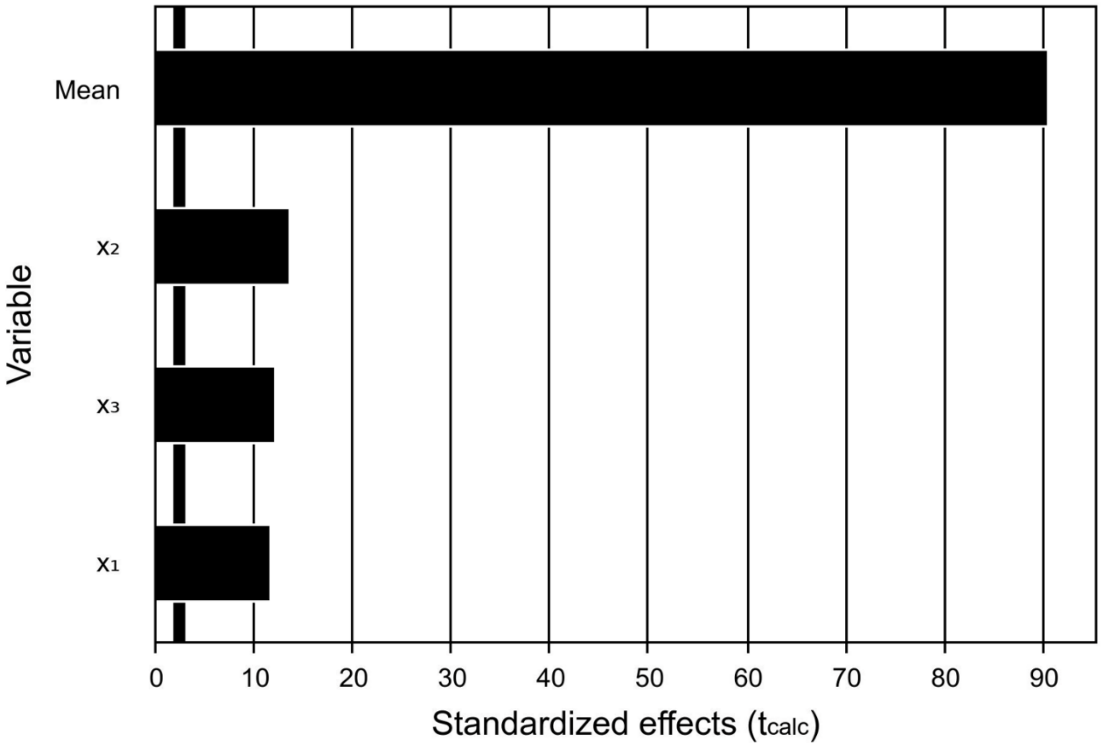

Figure S1. Pareto chart with the significant effects of the independent variables studied on the enzymatic synthesis of geranyl benzoate in a solvent-free system (*p*<0.05). X_1 represents the molar ratio, X_2 represents the temperature, and X_3 represents the enzyme amount. The experimental data and conditions are shown in Table 1.

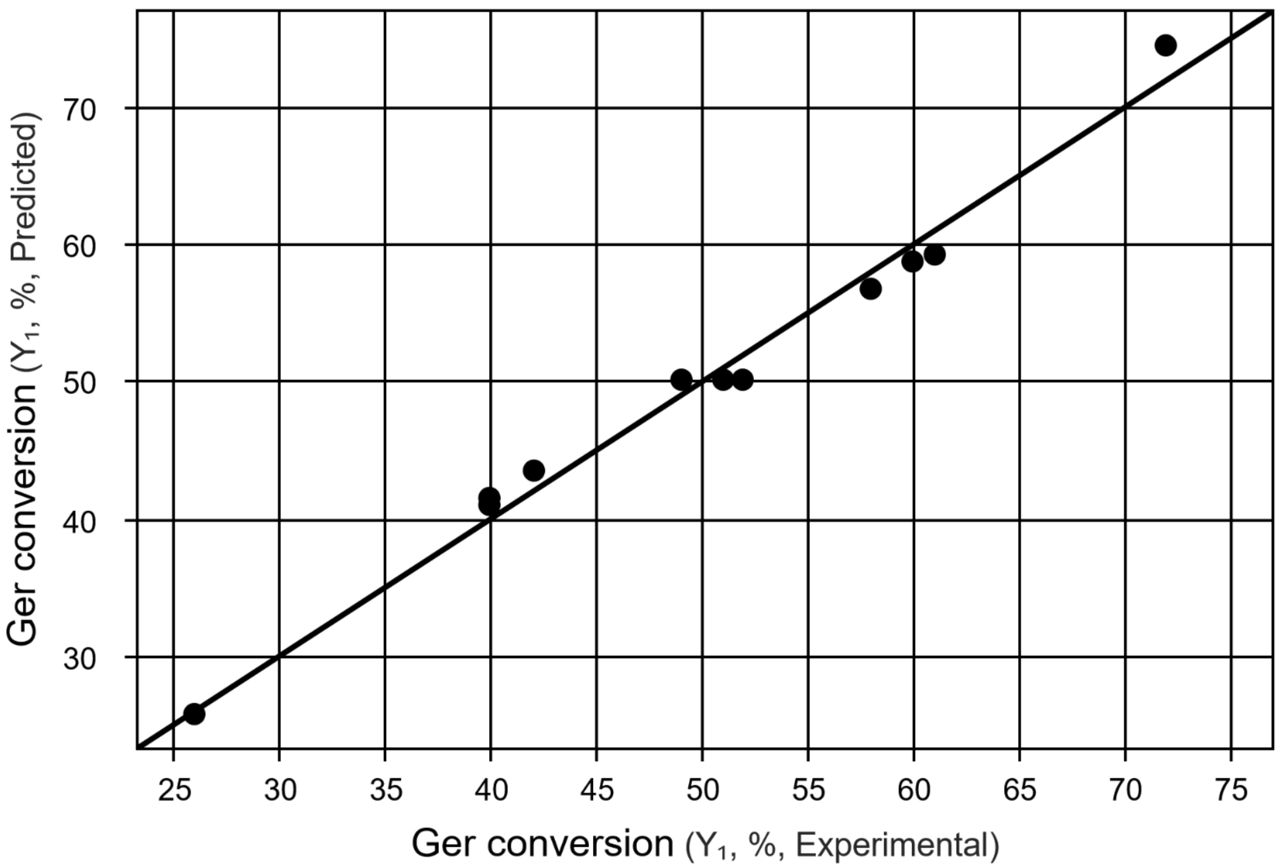

Figure S2. Experimental versus predicted geraniol conversion for the enzymatic synthesis of geranyl benzoate.

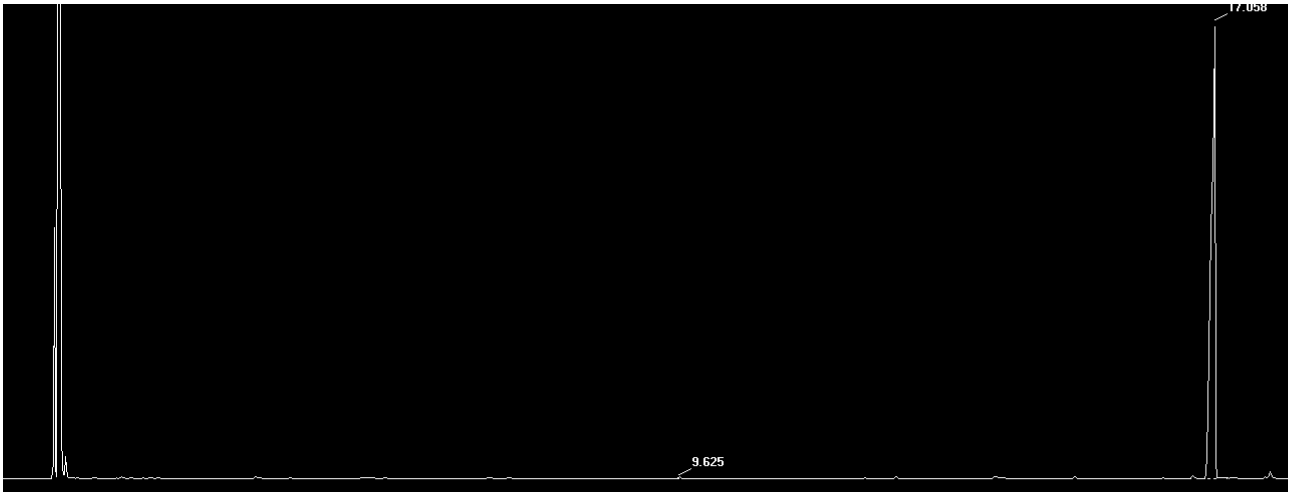

Figure S3. Typical gas chromatogram of geranyl benzoate after removal of the excess of methyl benzoate by vacuum distillation (rotary evaporator, 70 °C, 200 rpm, 10 mBar). Retention time: geraniol 9.62 min, and geranyl benzoate 17.05 min.

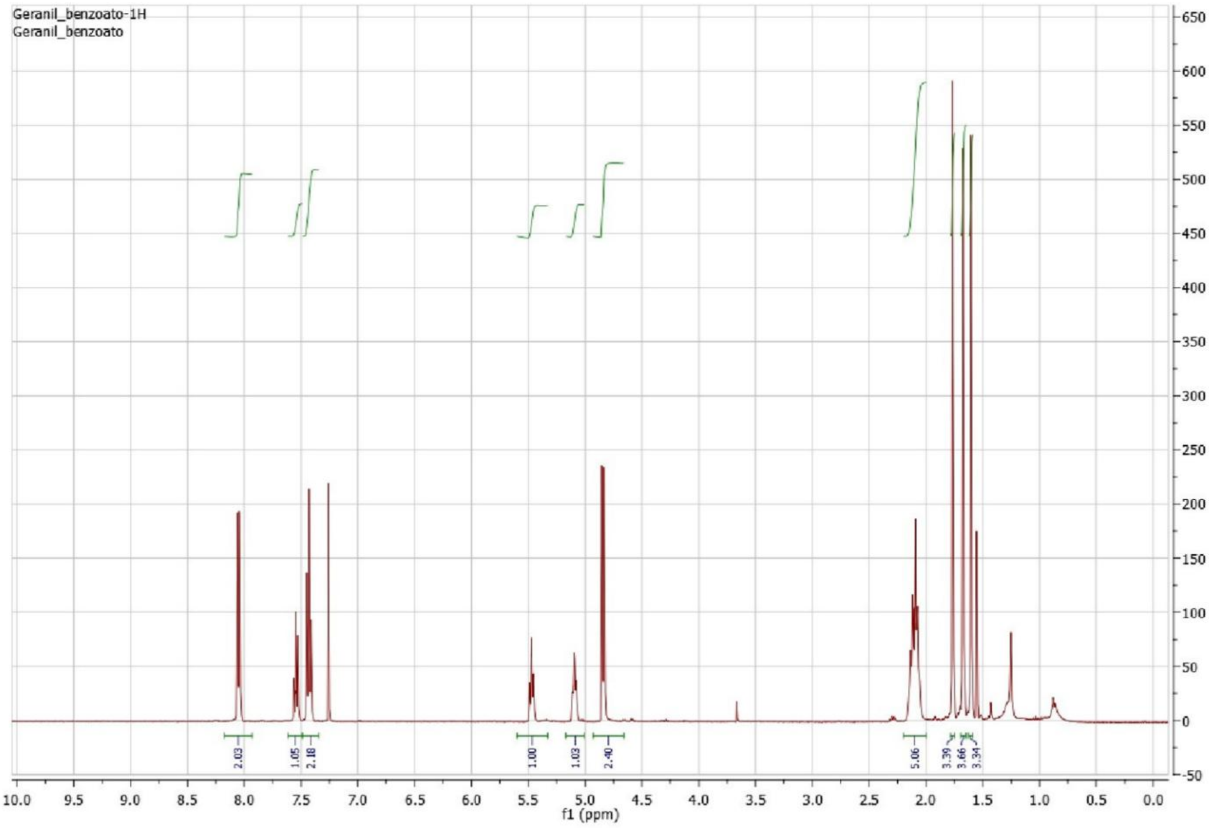

Figure S4. ^{1}H-NMR spectra of geranyl benzoate.

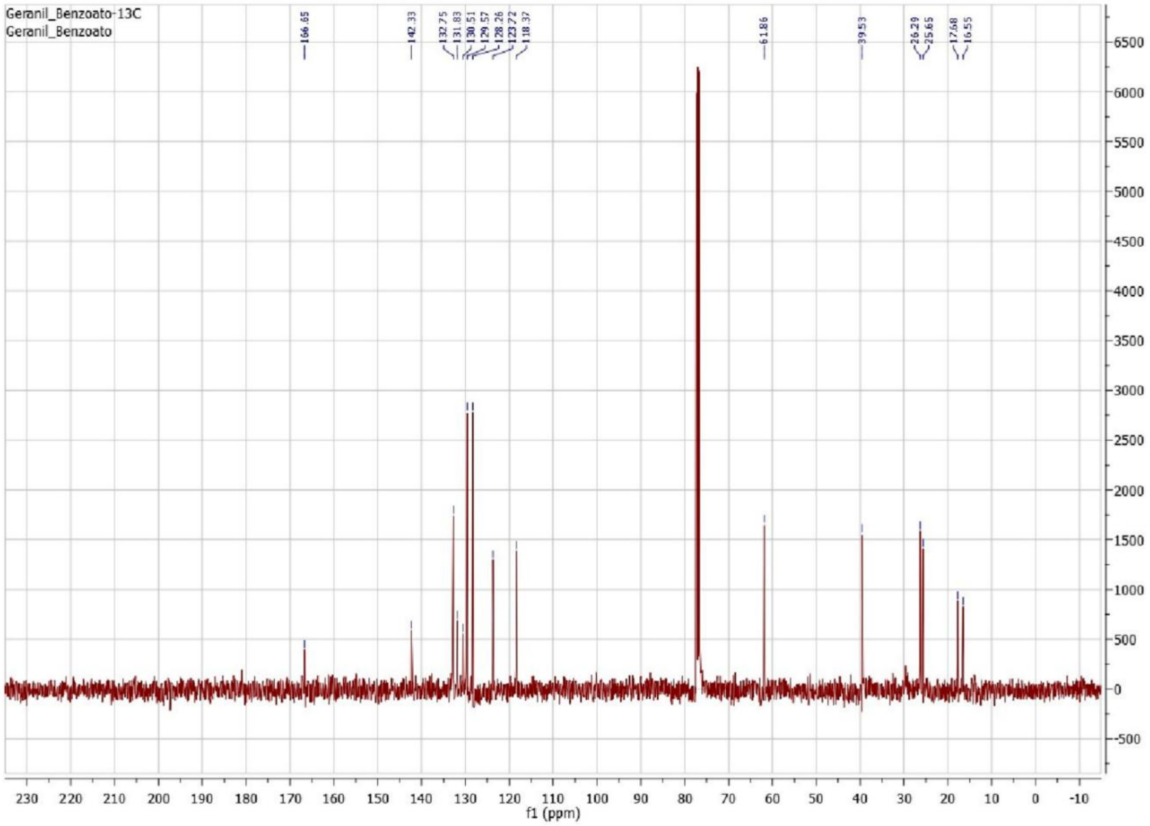

Figure S5. ^{13}C-NMR spectra of geranyl benzoate.
